# Preparing tomorrow’s medical specialists for participating in oncological multidisciplinary team meetings: perceived barriers, facilitators and training needs

**DOI:** 10.1186/s12909-022-03570-w

**Published:** 2022-06-27

**Authors:** Janneke E. W. Walraven, Renske van der Meulen, Jacobus J. M. van der Hoeven, Valery E. P. P. Lemmens, Rob H. A. Verhoeven, Gijs Hesselink, Ingrid M. E. Desar

**Affiliations:** 1 Department of Medical Oncology, Radboud University Medical Center, Postbus 9101, huispost 415, 6500 HB Nijmegen, The Netherlands; 2 Department of Research and Development, Netherlands Comprehensive Cancer Organization, Goldebaldkwartier 419, Utrecht, DT 3511 The Netherlands; 3 Department of Medical Oncology, Cancer Centers Amsterdam, Amsterdam UMC, University of Amsterdam, Meibergdreef 9, 1105 AZ Amsterdam, The Netherlands; 4 Department of Intensive Care, Radboud Institute for Health Sciences, Radboud University Medical Center, Postbus 9101, huispost 707, 6500 HB Nijmegen, The Netherlands

**Keywords:** Medical residents, Education, Collaboration, Communication, Clinical learning environment, Multidisciplinary team meeting, Oncology, Teaching roles, Training suggestions, Medical specialists

## Abstract

**Introduction:**

The optimal treatment plan for patients with cancer is discussed in multidisciplinary team meetings (MDTMs). Effective meetings require all participants to have collaboration and communication competences. Participating residents (defined as qualified doctors in training to become a specialist) are expected to develop these competences by observing their supervisors. However, the current generation of medical specialists is not trained to work in multidisciplinary teams; currently, training mainly focuses on medical competences. This study aims to identify barriers and facilitators among residents with respect to learning how to participate competently in MDTMs, and to identify additional training needs regarding their future role in MDTMs, as perceived by residents and specialists.

**Methods:**

Semi-structured interviews were conducted with Dutch residents and medical specialists participating in oncological MDTMs. Purposive sampling was used to maximise variation in participants’ demographic and professional characteristics (e.g. sex, specialty, training duration, type and location of affiliated hospital). Interview data were systematically analysed according to the principles of thematic content analysis.

**Results:**

Nineteen residents and 16 specialists were interviewed. Three themes emerged: 1) awareness of the educational function of MDTMs among specialists and residents; 2) characteristics of MDTMs (e.g. time constraints, MDTM regulations) and 3) team dynamics and behaviour. Learning to participate in MDTMs is facilitated by: specialists and residents acknowledging the educational function of MDTMs beyond their medical content, and supervisors fulfilling their teaching role and setting conditions that enable residents to take a participative role (e.g. being well prepared, sitting in the inner circle, having assigned responsibilities). Barriers to residents’ MDTM participation were insufficient guidance by their supervisors, time constraints, regulations hindering their active participation, a hierarchical structure of relations, unfamiliarity with the team and personal characteristics of residents (e.g. lack of confidence and shyness). Interviewees indicated a need for additional training (e.g. simulations) for residents, especially to enhance behavioural and communication skills.

**Conclusion:**

Current practice with regard to preparing residents for their future role in MDTMs is hampered by a variety of factors. Most importantly, more awareness of the educational purposes of MDTMs among both residents and medical specialists would allow residents to participate in and learn from oncological MDTMs. Future studies should focus on collaboration competences.

**Supplementary Information:**

The online version contains supplementary material available at 10.1186/s12909-022-03570-w.

## Introduction

The ultimate goal of medical education for residents (i.e. qualified doctors in training to become a medical specialist, also known as registrars) is that they become medical experts able to integrate competences such as communication and collaboration into high-quality – frequently multidisciplinary – patient care [1, 2]. Many complex diseases require a multidisciplinary team to establish the diagnostic or treatment trajectory. Within oncology, formal weekly multidisciplinary team meetings (MDTMs) are the standard of care in diagnosing, staging and determining the treatment strategy, which is often multidisciplinary. In such an MDTM committed surgeons, medical and radiation oncologists, radiologists, nuclear radiologists and pathologists work together, as do their residents. In addition, an administrator and a clinical nurse specialist (CNS) are also present [3, 4]. There are separate tumour-specific MDTMs for different types of cancer, e.g. uro-oncological or breast cancer MDTMs [5]. The duration of the meetings varies, usually between 1 and 2 hours, with an average of 2 minutes discussion time per patient [6].

Although the primary goal of the MDTM is to improve patient care, it is also a training opportunity for the residents present, both in terms of medical content as well as learning to collaborate competently and communicate with specialists of a different specialty. It involves learning in a clinical learning environment (CLE) as described by Nordquist et al. (2019), who state that learning in a clinical context is fundamental to the training of health professionals, as there is simply no alternative [7]; this also applies to participation in MDTMs. The CLE differs every few months since residents rotate internships, including the MDTMs involved.

While the importance of multidisciplinary collaboration is widely recognised, debate continues about how to acquire and measure these competences [8, 9]. Residents are expected to learn to participate within MDTMs according to the master-apprentice principle, in which they learn ‘on the job’ by observing the medical specialists and assume increasingly active participative roles in discussions during their training [10]. This implies that the medical specialists function as good role models [7, 10]. However, most of the medical specialists participating in oncological MDTMs are not trained to work in a multidisciplinary team (MDT). In addition, formal training programmes for interdisciplinary collaboration are absent in most countries [11]. Fahim et al. (2020) found that the decision-making process within MDTMs is negatively affected by the lack of soft skills, such as communication, collaboration and leadership [12]. An open and safe team climate is needed, but is not evident in the hierarchical MDTM setting that still exists in many teaching hospitals [13–15]. That hierarchy plays a role in MDTMs is also apparent from the often unseen role of the CNS, whose participation in the discussions is minimal, despite the fact that they could provide valuable information about the patient’s perspective [16, 17]. The CLE should provide role models, space and focus for residents to be able to actually learn how to participate competently in MDTMs [7].

Currently, there is a lack of understanding of residents’ needs with regard to learning to collaborate in a multidisciplinary manner in the CLE of MDTMs. Moreover, there is no evidence in the literature as to whether the MDTM as a CLE is an adequate method of teaching competent multidisciplinary collaboration. Hamoen et al. (2021), found that another CLE – the hospital ward – is centred around patient care rather than around education, leading to suboptimal multidisciplinary learning [18]. We wonder whether this also applies to MDTMs in general, and to oncological MDTMs in particular.

We therefore aimed to identify barriers and facilitators, as perceived by residents and medical specialists within the MDTM CLE, regarding the optimal preparation of residents for future competent MDTM participation and subsequent additional training needs.

## Methods

### Study design

A qualitative semi-structured interview study was conducted in the period between May 2018 and May 2019 in accordance with the Standards for Reporting Qualitative Research (SRQR) *(Appendix 1).* The local ethics committee (CMO region Arnhem – Nijmegen) approved the study (registration number ECSW-LT-2021-11-19-79,410). All participants received written information about the project and its aims, and agreed to participate.

### Participants

Interviewees were purposively sampled [19] in order to maximise variation in participants’ demographic and professional characteristics, using seven criteria: 1) regular participation in oncological MDTMs; 2) specialty (surgical, medical and radiation oncology, radiology, nuclear radiology, and pathology); 3) sex; 4) medical specialists versus residents; 5) training duration of residents (≤3 versus 4–6 years); 6) hospital region (coded to A-B-C-D, based on the provinces in the Netherlands) and 7) type of hospital (peripheral or academic). Interviewees were invited by e-mail to participate in our study by two researchers (JW and ID). After consent was obtained, an appointment was made.

### Data collection

Semi-structured telephone interviews were conducted by a medical oncologist (JW) who has been attending two MDTMs per week for 5 years, including 2 years as resident. Prior to the study, JW received interview training from an experienced researcher in the field of qualitative research (GH). JW had no personal relationships with the interviewees. Interviews were conducted using a topic guide, which was evaluated after each interview and adjusted if necessary. The main topics that guided question development were: 1) current experiences with (guidance of) participation of residents in MDTMs; 2) perception of the MDTM atmosphere as a working/learning environment and 3) thoughts on and suggestions for improving IPE and/or competence training for MDTM participation (Appendix B).

During the interview, JW used probes, summarised statements and took notes to fully comprehend and validate the perspectives of interviewees. All interviewees gave their consent prior to the start of each interview and at the end of each interview they were asked whether the information obtained was accurate and valid and whether they had any additional comments regarding what was discussed.

All interviews were audiotaped after obtaining interviewee consent and transcribed verbatim. Interviews lasted between 27 and 72 minutes, with a median duration of 38.7 minutes. The transcripts were loaded and stored on the computers of the hospital where the researchers work, using ATLAS.ti software version 8.0 (ATLAS.ti Scientific Software Development Company, Germany), a software program for detailed coding in qualitative data analysis.

### Data analysis

The data were analysed through thematic analysis, with the unit of analysis being the recorded interviews. In thematic analysis, researchers familiarise themselves with the data by reading and re-reading, generate initial codes, search for overarching themes and review these themes [20]. Three researchers (JW, RM, AO) were involved in reviewing and analysing the interview transcripts. The backgrounds of RM and AO were different to that of JW to ensure varying reflexive positions (RM = medical biology research student, AO = health scientist). Relevant data were identified and structured by open, axial and selective coding. Coding is the interpretative process in which conceptual labels are given to the data [21]. At first, all three researchers independently read the transcripts and coded relevant fragments to minimise the subjectivity of findings (open coding). After each interview, the transcript was coded before the next interview took place. During the iterative analysis process, researchers frequently shared and discussed the meaning and uniqueness of generated open codes. After discussion, codes were reformulated and those with the same meaning were grouped into one unique code (axial coding). After the open and axial coding of the first 15 interviews, all three researchers reached consensus on a list of codes (codebook) that guided the further coding of the rest of the interviews performed by one researcher (RM). New codes and related text fragments were then discussed with at least one of the other researchers. Finally, in the last transcripts only data that provided additional insights were coded (selective coding). Data sufficiency was reached after 35 interviews: i.e. new data no longer provided additional insights relative to the research question [22]. Throughout the analysis JW grouped codes belonging to the same concept into categories and finally identified themes from the data in consultation with other research members involved (ID, GH, RV). Data analysis was supported with the use of a qualitative data analysis software program (Atlas.ti version 8.0).

## Results

Thirty-five individual semi-structured interviews were analysed; 19 residents and 16 medical specialists participated. Interviewees were evenly distributed across genders and medical specialisations. More interviewees, especially residents, were located in academic hospitals (*n* = 23) than in peripheral hospitals (*n* = 12), reflecting the educational role of academic hospitals. The distribution of interviewees across the four regions in the Netherlands differed slightly. Most residents had already completed over 3 years of training (*n* = 15 versus *n* = 4). All residents initially had an observing role in MDTMs. During training their role shifted to presenting patients (for clinical specialties) (*n* = 8), describing the results of diagnostics (for diagnostic specialties) (*n* = 9), participating in discussions (*n* = 5) or taking minutes (*n* = 3) *(*Table 1*)*.

**Table 1:** Characteristics of participants

	Residents (*n* = 19)	Medical specialists (*n* = 16)
Gender		
Male	8	9
Female	11	7
Medical specialty		
Medical oncology	3	3
Radiation oncology	3	3
Surgical oncology	4	4
Radiology	3	2
Nuclear radiology	2	1
Pathology	4	3
Hospital		
Academic	16	7
Peripheral	3	9
Region^a^		
A	1	3
B	7	7
C	2	2
D	9	4
Training duration of residents (years)		
≤ 3	4	
4–6	15	
Residents role in oncologic MDTMs^b^		
Observer role initially	19	
Describing diagnostics	9	
Presenting patient cases	8	
Active participation in discussions	5	
Taking minutes	3	

^a^Regions are coded based on the provinces in the Netherlands

^b^*MDTMs* multidisciplinary team meetings

Three themes emerged from the analysis: 1) awareness of the educational function of MDTMs among medical specialists and residents; 2) characteristics of MDTMs and 3) team dynamics and behaviour *(*Table 2*).* In addition, Table 2 also lists the associated nine categories and 23 facilitators and barriers, including supporting quotes.

**Table 2:** How residents learn to participate competently in multidisciplinary team meetings: perceived facilitators and barriers

Theme	Category	Perceived facilitator (f) or barrier (b)	Representative quotes
Awareness of the educational function of MDTMs among medical specialists and residents	Both medical specialists and residents acknowledge the educational objectives of MDTMs*	Education is an explicit goal of the MDTM (f)	*Specialist [MO1]:* “First, the MDTM must be described as a training moment. Second, learning objectives must be established and these goals must be acted upon during the meeting.” *Specialist [S4]:* “If you do not pay attention to the education of residents, they will not learn. This also applies to their MDTM participation.”
		Non-medical competences are largely ignored (b)	*Resident [S1]:* “Participating in MDTMs is not incorporated in my educational programme. Ridiculous actually (…) The feedback I received is solely focused on the medical content and not on other competences.” *Specialist [S4]:* “Focus on the medical content is not enough (…) Focus on guiding residents on how to participate in MDTMs is the only way they can learn.”
	Residents’ self-study methods	Residents copy behavioural styles of their supervisors (f)	*Resident [MO3]:* “I watch how my supervisors conduct the MDTM. I copy their behaviour because I assume that is how it should be done.” *Specialist [R2]:* “I tell my residents that is important to see how I do the MDTM. Watch and learn. Then they can do the same.”
		Residents accept their responsibility to be well prepared (f)	*Resident [R9]:* “I need to prepare for the meeting in order to understand what is important to discuss and what is expected from me.” *Resident [S4]:* “If I have prepared well, I can pay full attention to all the information presented during the meeting and I can change my predefined treatment proposal. If I am not prepared, I usually can’t switch right away and my learning curve is much less.”
	Educational conditions in MDTMs	Residents need time for observation to learn (f)	*Resident [RO1]:* “For now, I am just observing. I did prepare each case in advance and devised a treatment proposal for myself. I learn a lot by listening to the discussion and finding out if my ideas were right or wrong.” *Specialist [MO3]:* “Residents need to observe (…) to learn to understand specialists’ considerations that lead to decisions.”
		Residents cannot take on an active role (b)	*Resident [MO1]:* “I just listened. In a few months I will complete my training and should be an active member at the front table (…) I don’t feel qualified for that since I haven’t been trained to do this.” *Resident [S4]:* “I tried a few times to give input, but it felt like I was being ignored, not taken seriously. It felt like I was not invited to participate in the discussion.”
	Teaching role of medical specialists	Supervisors help residents prepare for the MDTM (f)	*Specialist [R1]:* “Before the meeting, I check how the resident has prepared for the MDTM and what they find important to discuss during the meeting. Then I can suggest adjustments.” *Resident [RO2]:* “Before each MDTM I discuss the cases that we present with my supervisor. We then formulate a treatment proposal in advance. I find that very useful, because I now know that I am speaking on behalf of us both during the MDTM.”
		Supervisor supports an active role for residents (f)	*Specialist [RO2]:* “I prepare and discuss all cases with the resident in advance. During the meeting I give my resident an opportunity to take on their role. The most difficult part for me is not to intervene.” *Specialist [N1]:* “We give residents more speaking time than we give ourselves (…) so they can learn the trade.”
		Supervisor provides back-up (f)	*Resident [P2]:* “My supervisor sits next to me at all times. It is a reassuring idea to have back-up if it gets too complicated for me.” *Resident [RO1]:* “When I do not know the answer, I look sideways at my supervisor.(…) That is why the supervisor is also present at the MDTM, to help me with these cases.”
		Supervisor gives feedback to residents (f)	*Specialist [MO]:* “I believe that residents learn through the master and apprentice principle. I want to be a good role model for them. Giving feedback on their performance is part of that role.”
Characteristics of MDTMs	Time constraints	Time constraints make it difficult for residents to ask questions or take part in the discussion (b)	*Resident [S3]:* “There is a lot of time pressure, with only two minutes discussion time per patient (…) This creates a barrier to asking for further explanation.” *Resident [RO1]:* Usually there is only time to discuss complex cases, which are too complicated for me.”
		MDTM is not a formal part of the residents’ educational programme (b)	*Resident [S1]:* “I cannot always attend MDTMs because of other daily tasks, even when my supervisor expects me to. (…) It would help if the meeting was booked in my schedule.” *Specialist [MO1]:* “Residents consider the MDTM as an extra task. (…) They feel pressure to perform well. (…) So they stay away from the MDTM if they haven’t been able to prepare for the meeting due to other daily tasks.”
	Regulations and organisation	Residents are seated in the front row / seated within the inner circle of an U-shaped setting (f)	*Resident [P1]:* “In the beginning I sat at the back (…) Now I try to sit at the table, because then you feel more involved. You can look other participants in the eye instead of looking at their backs.” *Resident [P3]:* “The previous MDTM room was very small. Then I had to sit at the back. Now we have a new, bigger room and I can sit at the front Table. I feel much more involved now. Saying something is easier.”
		There is an assigned responsibility for residents in the division of tasks (f)	*Specialist [N1]:* “Usually, residents prepare and present the cases. Only in complex cases do specialists take over. (…) We encourage them to propose a treatment plan as well.” *Resident S2:* “My task is to present the cases. I have learned how I am supposed to do that; what information I share and what is not necessary.”
		Residents are responsible for tasks that hinder active MDTM participation (b)	*Resident [S3]:* “I was expected to take the minutes and participate actively at the same time, but I was not able to concentrate on the content.” *Resident [S1]*: “At one MDTM I had to chair, present cases and take the minutes. Sometimes supervisors would ask me what my treatment plan was. Most of the time I could not answer because I had too much on my plate.”
		The participation period of residents in one tumour-specific MDTM is too short (b)	*Resident [RO1]:* “Every six months I focus on a different tumour type with its own tumour-specific MDTM. (…) So, every time I start to feel comfortable with getting a bigger role in the MDTM, I switch to another tumour type and it starts all over again.” *Resident [MO3]:* “I am now doing my colorectal internship for 6 months. It takes me time to acquire the right amount of knowledge to participate in discussions and to know what is expected of me in this particular MDTM. By the time I have taken on a more active role, my internship is almost over.”
Team dynamics and behaviour	Atmosphere and hierarchy within MDTMs	The MDTM lacks an open and friendly atmosphere (b)	*Resident [RO3]:* “Specialists can communicate quite violently with each other. (…) Communication training for the core team members might help them understand how a resident feels in this setting.” *Specialist [P3]:* “One of the specialists grumbles regularly, although he means well. (…) At first, it can be quite threatening.”
		The hierarchal position of residents hinders their MDTM participation (b)	*Resident [MO3]:* “Due to my position as a resident, it is difficult for me to express an opposing opinion to a specialist.” *Specialist [MO2]:* “There is always one participant, usually a professor, who is in charge and who has the final say. I believe that younger participants should be given more space to express different opinions.”
		Residents are not seen as full team members (b)	*Resident [RO3]:* “I have the feeling that they (medical specialists) doubt everything I say. Especially because I sometimes see them email each other afterwards.” *Resident [N1]:* “When an older and therefore more experienced radiologist says something, all other members automatically listen to him and not to what I say. Even if I am strongly convinced that I am right.”
		Residents are not familiar with the other team members (b)	*Resident [MO2]:* “I feel more free to speak if I have been to the MDTM multiple times, because then I am more familiar with the team and feel part of the team.” *Resident [P2]:* “I was very nervous about attending the MDTM. I stepped into a large room with many screens and a group of people that I do not know. (..) I thought: ‘O my god, this is never going to work’.”
	Creating a safe and open learning environment	There is scope to make mistakes (f)	*Specialist [MO1]:* “If the resident says something that I think is not quite right, I give them the space to make their arguments. Then I say: ‘Maybe there is another option …. I feel this is more respectful than interrupting right away.” *Resident [RO]:* “If I say something that my supervisor thinks is not quite complete, she takes over. Then I cannot take back control.”
		Residents are continuously assessed on their functioning (b)	*Resident [S3]:* “The setting of the meeting feels like an exam (…). It creates a barrier preventing me from speaking.”
	Residents’ personal characteristics	Resident lacks self-confidence and assertiveness (b)	*Resident [P3]:* “Sometimes I do not dare to speak, because then I think: ‘I am just a resident, what do I know? (…) I am too shy.” *Resident [P4]:* “To be secure in drawing conclusions, you need to have the medical knowledge, but also the confidence in yourself that you can communicate within the team. (…) I am assertive, but I know that other residents have more of a struggle.”

[S] = surgical oncologist, [MO] = medical oncologist, [RO] = radiation oncologist, [R] = radiologist, [N] = nuclear radiologist, [P] = pathologist

*MDTM = Multidisciplinary team meeting

### Theme I: Awareness of the educational function of MDTMs.

Four categories were identified within this theme: 1) acknowledgment by both medical specialists and residents of the educational objectives of MDTMs; 2) residents’ self-study methods; 3) educational conditions within MDTMs and 4) teaching role of medical specialists.

#### Acknowledgment by both medical specialists and residents of the educational objectives of MDTMs

All interviewees agreed that MDTMs not only serve patient care but also have educational goals, primarily to increase medical knowledge. Collaboration and communication competences as additional educational goals were seldom mentioned spontaneously, although acknowledged after questioning. Several interviewees stated that recognition of all the educational aspects of MDTMS by both medical specialists and residents is a prerequisite for an optimal learning environment.

#### Residents’ self-study methods

Some residents remarked that it is their responsibility to acknowledge competent MDTM participation as a learning objective. Medical specialists agreed, and expected residents to have their own plan of action to achieve this goal. Residents stated that to achieve this learning objective, they mainly rely on intuition and observation of medical specialists who serve as teaching models, confirming the master-apprentice principle. Residents copy the behaviour of their supervisors, assuming they have already learned the skills to participate effectively in an MDTM.

Medical specialists and residents both believe it is the resident’s responsibility to prepare for the MDTM. Many residents indicated that if they do not prepare, participation in the MDTM is much less valuable because, unlike medical specialists, they cannot yet fall back on experience and substantive knowledge. Some residents said that if they were not prepared, they felt insecure and would rather not be present at all.

#### Educational conditions within MDTMs

Residents indicated that in their first period of MDTM participation, an observer role helps them determine whether their medical knowledge is sufficient, which encourages them to take the next step towards active participation. Medical specialists agreed that a resident without sufficient medical knowledge cannot participate.

Residents stated that practicing being an active team member within the MDTM helps them to grow into their future role. However, some residents struggled to take an active role, as they felt unheard or were not given the opportunity for active participation.

#### Teaching role of medical specialists

Many interviewees expressed the importance of medical specialists recognizing their teaching role and acting accordingly. This is a facilitator for an open and safe atmosphere and offers residents scope to ask questions. Residents indicated that a good supervisor gives feedback, prepares difficult patient cases together with the resident, offers them the opportunity to take an active role by giving them the opportunity to speak during the MDTM and provides back-up where necessary. Medical specialists mentioned that they prefer to provide this back-up by sitting at the table next to the resident. Residents recognised the sense of security offered by such a side-by-side set-up, but also indicated that it creates a barrier to speaking, as other team members are likely to focus on the medical specialist rather than the resident. Medical specialists recognise this and say they dominate the discussion out of enthusiasm, rather than because of suspected incompetence of residents.

### Theme II: characteristics of MDTMs

Two categories were identified within this theme: 1) time constraints and 2) regulations and organisation.

#### Time constraints

The barrier most spontaneously mentioned by interviewees was time pressure. Residents reported that they were reluctant to ask questions about cases, because they were unwilling to disrupt the rapid flow of case presentations. Interviewees indicated that, under time pressure, the focus of the MDTM automatically tends to the medical content and there is less concern for educational aspects. It was seen as the chair’s responsibility to strike a balance between spending sufficient time on medical content and meeting residents’ needs.

Residents reported that they have a busy schedule with direct and indirect patient-related tasks. The first has priority over the second, which means that they sometimes have insufficient time to prepare properly or to attend the MDTM at all.

#### Regulations and organisation

In the Netherlands, each hospital has its own MDTM regulations and organisation. These regulations include the physical location of members in the room, the order in which members should speak and who takes the minutes. The regulations regarding educational aspects of the MDTM are unknown to most of the interviewees. Some residents indicated that they were clearly given the task of initiating the patient’s case or presenting the outcomes of diagnostics, which automatically made it easier for them to take on a more active role. However, they indicated that this does not mean they can actually take the step of participating in the discussion about the patient case as well.

Some residents mentioned they are seated at the back, while others are in a U-shaped arrangement at the front. Residents found the latter facilitated an active role. Where in most hospitals the minutes are taken by secretarial support staff, in some hospitals this is a task for residents. This was felt to interfere with active participation within the MDTM and its educational goals.

Residents in clinical oncological specialties, such as surgery, medical and radiation oncology, who have tumour-specific internships that rotate every few months, indicated that they did not feel competent enough to participate in discussions until the end of such an internship. After a rotation to a different tumour-specific MTDM, they felt the need to start again as a listener instead being an active team member. This was partly due to lack of knowledge and partly due to unfamiliarity with the team.

### Theme III team dynamics and behaviour

Three categories were identified within this theme: 1) atmosphere and hierarchy within MDTMs; 2) creating a safe and open learning environment and 3) residents’ personal characteristics.

#### Atmosphere and hierarchy within MDTMs

Residents benefit from an open and friendly team atmosphere since it removes barriers to active participation. However, the atmosphere can also be too friendly, according to a medical specialist who said that too many jokes distract from the medical content. In contrast, in a hierarchical structure of relations, dominant team members leave little room for residents to voice their opinions. A number of residents expressed a feeling that medical specialists did not regard them as full members of the team, reject their input in discussions and only accept input from more experienced medical specialists.

Medical specialists indicated that team atmosphere in MDTMs improves when team members feel connected. Residents switching from one tumour-specific MDTM to another feel that they have to prove themselves over and over again. They lack the sense of belonging to the team, which makes them nervous and reluctant to talk.

Some interviewees stated that communication between two or three team members could come across as quite aggressive, with lengthy discussions and no compromise. Residents indicated that the barrier to getting involved in such a discussion was perceived as high. Mediation by a supervisor or chair was found to be the most effective way to address this behaviour.

Time pressure in MDTMs also affects the atmosphere; residents feel that hasty team dynamics make it more difficult for them to participate in discussions.

#### Creating a safe and open learning environment

Acknowledging the educational function of MDTMs is an important step towards creating a safe and open learning environment, according to interviewees. The fact that residents are still learning should be taken into account, so that mistakes can be made without immediately resulting in an uncomfortable or tense atmosphere. Residents indicate that feeling they are being continuously tested, as if they are taking an exam, makes them reluctant to participate actively in the MDTM.

#### Residents’ personal characteristics

The extent to which residents feel hindered in active participation is also believed to depend on personal characteristics: namely the degree of self-confidence, and whether the person is shy or assertive. Personal doubts regarding their own level of medical knowledge or communication skills hampered some residents in their MDTM participation. Lack of self-confidence was mentioned only by residents who also described themselves as introverted or shy. More assertive residents did not say they felt insecure.

### Additional training suggestions

Some residents indicated that the current method of education – listen only at first, and contribute actively to discussions when more experienced, with an emphasis on gaining medical knowledge – fails to make residents feel fully competent to participate in future MDTMs. Some medical specialists, especially those who have recently qualified (< 5 years), confirmed that they did not feel completely competent initially. Both medical specialists and residents described the need to better recognise and harness the educational potential of MDTMs with regard to collaboration and communication competences. However, not all interviewees felt the need for additional competency training for themselves. Nor did they spontaneously mention the educational value of MDTMs in learning to collaborate and communicate with specialists of a different specialty.

Suggestions given to improve the MDTM training programme mainly focused on the communication skills of the MDT, optimising the meeting atmosphere and addressing undesirable behaviour *(*Table 3*)*.

**Table 3:** Additional training suggestions, given by interviewees, to participate competently in multidisciplinary team meetings

Simulation MDTM* for residents only (safe)	
Simulation MDTM for all participants (change patterns)	
Behavioral styles, communication and collaboration skills training	
Efficient meeting skills training	
Video-recording of MDTM, independent observer feedback	
Training on pitching (medical) information	
Presence of an external behavior observer for feedback to all participants	
Mainly residents speak, supervisors only if necessary/asked	

*MDTM = Multidisciplinary team meeting

MDTM-simulation training was frequently referred to, because of the opportunity to pause at any moment for evaluation. Some interviewees stated that such simulation training should be organised for all MDT members and not just residents, otherwise medical specialists would maintain old habits. They believed it could increase awareness of patterns and behaviours and thereby improve team climate. Others mentioned as a possible disadvantage of such training that it could be perceived as unsafe, especially in a MDT with a distinct hierarchy. Instead of simulating MDTMs, video recording MDTMs to discuss collaboration and communication in retrospect was also suggested.

Other interviewees suggested more general training courses. Areas to be covered should include behavioural styles, communication types, meeting skills and pitching.

For all training suggestions, an external observer was recommended, to ensure an open and safe learning environment.

## Discussion

A variety of factors both hamper and support the way residents currently learn about competent participation in Dutch oncological MDTMs. This study identified the following facilitators: both medical specialists and residents acknowledging the educational function of MDTMs beyond their medical content and supervisors acting as teaching models, creating a safe and open learning environment and enabling active participation of residents. Barriers included time pressure, hierarchy, unfamiliarity with the team, regulations, an organisational structure that interferes with active participation and inhibiting personal characteristics of residents.

Lack of time compels MDTMs to adopt a business-like atmosphere with a structured sequence of medical specialists speaking [13, 23]. Along with hierarchy this can create barriers to speaking up [24, 25]. This was also found in an observational study in Germany (2015), which analysed the discussion process in 15 MDTMs. They concluded that junior physicians not only have a non-prominent role, but also that decisions were made only by senior physicians [26]. We also found that some residents did not feel completely free to speak. Reported reasons for this were perceived lack of knowledge, personal characteristics, hierarchy, and time constraints. Time pressure is a known problem in oncological MDTMs due to more complex treatment strategies and increasing numbers of patients to be discussed. Restructuring the organisation of MDTMs, for instance by selecting complex patient cases rather than discussing each patient with cancer, might offer time for explanations of difficult cases and create scope for residents to play a more active role [27, 28].

A review of the learning process of residents reported that they see their supervisors as role models and imitate their behaviour [29]. In this study, residents also described copying the behaviour of medical specialists, even when these supervisors were untrained and when team dynamics were influenced by hierarchy. In the literature, hierarchy is known to negatively influence teamwork and learning processes in the operating theatre [30, 31] and emergency department [32]. Our study showed that hierarchy also affects residents’ learning ability in oncological MDTMs, although the degree of impact also depends on the personal characteristics of the resident. Being shy and introvert may interfere with optimal performance [33]. Medical specialists should recognise these personality traits and encourage residents to play a more prominent role in MDTMs. Suggested training options in the field of behavioural styles, communication or pitching skills can be offered on an individual basis and should focus on personal features as well as on recognising patterns in other MDTM participants.

We believe that the training suggestions given by the interviewees – simulating or video recording MDTMs – might improve team skills, expand medical knowledge and allow scope for questions without participants feeling rushed. It could be an excellent opportunity to teach residents specific MDTM skills, such as learning to discuss a person who is not your patient, but for whom you take responsibility. Furthermore, aspects of teamwork such as shared understanding, mutual support and psychological safety could be highlighted in such training [34]. Although further studies need to be conducted to demonstrate the added value, it is likely that not only residents but the entire MDT would benefit from such training. In addition, training can be conducted in a broader perspective than the oncological MDTM, as MDTMs exist in many other areas of health care as well.

### Limitations

Our findings need to be interpreted in the light of several limitations. First, we aimed at a diverse pool of interviewees, but found some inequalities. Most residents were from academic hospitals, which reflects the educational role of academic hospitals. However, some residents had already completed an internship in a peripheral hospital and were also able to include their experiences from those internships during the interviews. Furthermore, most of the residents had had more than 3 years of training. This is because residents in clinical oncological specialties are only present at MDTMs during their final years of training due to specialisation.

Second, our study was conducted solely in the Netherlands. Cultural differences may influence MDT functioning, e.g. hierarchy. There may also be some differences in the educational programme followed by residents in MDTMs. Nevertheless, we believe that our general findings are relevant worldwide, as MDTMs are common practice and not specific to oncology.

Third, interview findings may be biased by the medical background of the primary researcher: this may have steered the direction of the interview or interpretation of data. However, this background enabled JW to empathise with the experiences and perceptions of the interviewees and to interpret them. In addition, JW received extensive interview training that made her aware of the potential impact of her background and enabled her to avoid subjective questions and socially desirable answers.

Lastly, we conducted telephone interviews, which may have imparted a different dynamic or depth compared to face-to-face interviews. By using the telephone we may have missed important non-verbal cues enabling us to probe further. However, we opted for telephone interviews to increase the opportunities for making an appointment with the interviewees (who have busy schedules), which may also have increased their willingness to participate. The interviewer was aware of the potential disadvantage of a telephone interview and paid extra attention to specific non-verbal cues such as silences in the conversation or a raised voice.

## Conclusion

A variety of factors currently hamper the way residents learn to participate competently within Dutch oncological MDTMs. Residents can be helped to prepare for their future role as specialists in MDTMs through acknowledgement of the educational function of MDTMs by both residents and medical specialists, adjusting MDTM characteristics that hinder residents’ active participation, solving time constraints and creating a safe and open learning environment. Future studies should focus on collaboration and communication competences and their influence on team performance in MDTMs.

## Supplementary Information

**Additional file 1.**

**Additional file 2.**

## Data Availability

The datasets used and/or analysed during the current study are available from the corresponding author on reasonable request.
