# Supplementary material for: Preparing tomorrow’s medical specialists for participating in oncological multidisciplinary team meetings: perceived barriers, facilitators and training needs
Source: BMC Med Educ. 2022 Jun 27;22:502. doi: 10.1186/s12909-022-03570-w (PMC9238222; doi:10.1186/s12909-022-03570-w)
Supplement: Supplementary file 2 — Additional file 2. [file 12909_2022_3570_MOESM2_ESM.docx]

**Appendix B. Topic guide for semi-structured interviews on facilitators, barriers and training needs among residents to competently participate in oncological multidisciplinary team meetings.**

1. Introduction
   - Introducing, explaining goal, asking for consent on recording
   - Verifying specialism, resident or specialist, affiliated hospital, number of years of training, number and type of tumour specific MDTM^*^ interviewee is participating in.
2. Current experiences with (guidance of) participation of residents in MDTMs
   - What is the current role of the interviewee in the MDTM? Has this changed? If yes, how? Are you always present at the MDTM? What are reasons for not being present?
   - How does interviewee prepare for the MDTM?
   - For residents: How is supervision arranged? Is there a preliminary meeting with the supervisor? How does the resident feel about the supervision? What goes well? Is there a need for improvement? If yes, what kind of improvement? Receiving feedback? What is the role of the supervisor during the MDTM? Have learning goals been established with regard to MDTM participation?
   - For medical specialists: How is their current teaching role arranged? What is helpful and what hinders?
3. Perception of the MDTM atmosphere as a learning/working environment
   - How is the MDTM atmosphere experienced? Is there hierarchy? Is yes, what effect has this on the interviewee? How is the learning climate experienced?
   - For residents: How do you experience the scope to speak? Confidence?
   - What hinders active participation in MDTMs? And what facilitates participation?
4. Educational role of MDTMs and future training needs
   - Do you see an educational role for the MDTM? Why yes/no?
   - How do you experience the educational function of MDTMs? What competences needs to be obtained? Are there MDTM – educational regulations?
   - How is the current way of training for MDTM participation arranged? Is there a formal training program for participating in MDTMs? Do you discuss learning goals for MDTM participation?
   - For residents: Do you feel that the current way of MDTM participation prepares you for your future role in MDTMs? If not, what is missing and why?
   - For medical specialists: Looking back, how did you experience your MDTM participation at the beginning? How do you experience your current MDTM performance? How do you see your current teaching role during MDTMs?
   - Is there a need for training programmes on MDTM participation? If yes, what should be the focus of such training? Do you have a suggestion on how to perform such training? What are the advantages / disadvantages?

^*^MDTM = Multidisciplinary team meeting
